# Internet Infrastructures and Health Care Systems: a Qualitative Comparative Analysis on Networks and Markets in the British National Health Service and Kaiser Permanente

**DOI:** 10.2196/jmir.4.3.e21

**Published:** 2002-12-31

**Authors:** Ann C Séror

**Affiliations:** ^1^Université LavalQuébecCanada

**Keywords:** Internet, telecommunications infrastructures, delivery of health care, telemedicine, qualitative research, ideology, national health programs, National Health Service, Kaiser Permanente

## Abstract

**Background:**

The Internet and emergent telecommunications infrastructures are transforming the future of health care management. The costs of health care delivery systems, products, and services continue to rise everywhere, but performance of health care delivery is associated with institutional and ideological considerations as well as availability of financial and technological resources.

**Objective:**

To identify the effects of ideological differences on health care market infrastructures including the Internet and telecommunications technologies by a comparative case analysis of two large health care organizations: the British National Health Service and the California-based Kaiser Permanente health maintenance organization.

**Methods:**

A qualitative comparative analysis focusing on the British National Health Service and the Kaiser Permanente health maintenance organization to show how system infrastructures vary according to market dynamics dominated by health care institutions ("push") or by consumer demand ("pull"). System control mechanisms may be technologically embedded, institutional, or behavioral.

**Results:**

The analysis suggests that telecommunications technologies and the Internet may contribute significantly to health care system performance in a context of ideological diversity.

**Conclusions:**

The study offers evidence to validate alternative models of health care governance: the national constitution model, and the enterprise business contract model. This evidence also suggests important questions for health care policy makers as well as researchers in telecommunications, organizational theory, and health care management.

## Introduction

The Internet and emergent telecommunications infrastructures are transforming health care management as well as the dynamics of health care service markets. The costs of health care delivery systems, products, and services continue to rise everywhere, but performance of health care delivery is associated with institutional and ideological considerations as well as availability of financial and technological resources [1-3]. Health is defined here as "a state of complete physical, mental and social well-being and not merely the absence of disease or infirmity" [4]. This definition extends beyond the context of the individual to include population health and quality of life as well as community well-being [5]. In this view, the social environment is considered the source of health and social functioning as well as disease and social problems [6].

The World Health Organization evaluation of the United Kingdom and US health care systems ranks the United Kingdom 18th and the United States 37th of 191 countries rated, while the United States spends 40% more per capita on health care [7-9]. Economic models of competitive markets have been demonstrated to be inadequate for understanding health care sector performance [10]. Research shows for example, that life expectancy, a commonly-accepted health care performance indicator, is only indirectly correlated with gross national product through variables related to equitable wealth distribution and investment in public health services [1]. Information and telecommunications technologies are changing the configuration and modifying the definition of health care system efficiency and effectiveness. While rapid access to medical information and expert consultation represents a very significant contribution to health care services, distribution of technological resources and the dynamics of information flows in health care markets are not symmetrical, posing a particular challenge to the design of sustainable health care management systems.

The objective of this paper is to identify ideological differences in health care market infrastructures, including the Internet and telecommunications technologies, by a comparative case analysis of two large health care organizations: the British National Health Service (NHS) and the California-based Kaiser Permanente health maintenance organization.

### The Internet, Telecommunications, and Health Care Systems

Growth of the Internet and of the telecommunications sector has affected management in all economic sectors including health care [11-15]. Convergence between enterprise intranets and the Internet contributes to development of services such as content distribution and access infrastructures directly offered on the Internet. Examples in the health care sector include e-mail services among health care professionals and patients, discussion and support groups, messaging and fax services, specialized information search, portal infrastructures, and Web hosting. Management of such services on the Internet reduces the need for specialized personnel such as Webmasters and network managers within traditional health care institutions. Emergence of software applications - ASPs (Application Service Providers) - and infrastructures on the Internet reduces software licensing as well as human resources and other administrative costs associated with local acquisition and management. Database management outsourced to Internet infrastructures reduces network complexity and overload while increasing the speed, reliability, and rigor of information searching and processing.

Examples of these health care infrastructures are apparent in the British model of social medicine at the National Health Service's NHSNet, where convergence between the system intranet and the Internet contributes to a culture of open information exchange. Commercial services on the Internet, such as Cymedix [16] and MedSeek [17] also offer integrated intranet and Internet infrastructures for health care providers, health plans, laboratories, and hospitals. Gateway information infrastructures further serve market supply-and-demand requirements at the national, institutional, professional, or consumer (search engine) levels of analysis [18]. Wireless application service providers are bringing Internet-leveraged services to health care while minimizing modification to physician behavior required for adoption of health care applications [19]. Beyond institutional and area-network boundaries, evolutionary pressures transform assumptions of relatively stable, homogeneous, and centralized systems. Such pressures for collaboration, data sharing, and access to distributed resources increase the focus on interconnection of services both within and across organizations. These services include intelligent networks, switching devices, caching services, appliance servers, storage systems, and storage-area network-management systems in computational and semantic grid architectures comprised of standard protocols, services, application programming interfaces, and software development tools enabling resource sharing. Thus both technological trends and commercial pressures foster service decomposition and distribution through networks rather than host-centric systems [14,15,20].

Medical errors often occur in the clinical communication space, and they may be associated with cognitive difficulties of processing complex information as well as limitations of human memory. Cognitive strategies for coping with such difficulties include development of selective schemata for retention of critical information [21] and use of asynchronous communication channels to manage memory overload in an interruptive communication environment [22]. The type of computational support or telecommunications infrastructure appropriate to clinical decision making may be associated with the common ground shared by human and technological agents in communication processes, as well as the context of such common ground [23]. Stable common ground shared by actors in the clinical decision-making system may be associated with asynchronous modes (prerecorded; store-and-forward) of information transmission, and active computational modeling based on preemptive information storage. For example, prefetching methods may be used to restore archived images to workstations in anticipation of needs for historical case comparisons, patient management, or clinical problem solving [24]. On the other hand, shifting ground may require isochronous (real-time) telecommunications transmission, and conversation through passive computing channels [23]. It is critical to note that real-time talk accounts for as much as 50% of clinical information transactions. This continues to be necessary in increasingly interdisciplinary and diverse medical teams despite availability of advanced information technologies for clinical decision support. Modeling clinical decision making in practice requires holistic analysis of human, software, and infrastructural components of both clinical decision making and human collaboration focused on goal achievement [25]. Cultural and ideological assumptions underlying work practice determine the dynamics of system-wide behavior [26-28].

Diverse models of service distribution and payment promote entry of new institutional actors into the health care system, while development of advanced telecommunications infrastructures may support either centralized health care authority or free-market dynamics driven by consumer demand [29]. Consumer participation is generally associated with system emphasis on preventive health care strategies while at the same time managerial tools are designed to control health care costs [28]. Limited health care resources may be associated with participative rationalization of health care services by systematic gathering and integration of individual and collective consumer preferences in methodological models for budgeting and other resource allocation.

**Table 1:** Health care markets: dominant control of structures and processes [29]

**Market Dynamics for Health Care Products and Services: Supply (Push) by Professionals**		
	**Control: Clan (Through Norms and Standards)**	**Control: Hierarchy (Through Institutional Infrastructures)**
	**Model: Professional Covenants**	**Model: National Constitution**
Structure:	Internet network	Proprietary network (WAN/LAN)
Architecture	Distributive multiagent system	Federation
Gateways	Professional and subject	Institutional
Integration	Associative clinical process (medical specialties)	Federative structural
Access control	By health care professionals	By institutional and telecommunications network structures
Authority	Professional	Centralized national hierarchies
Ownership	Diverse and stable market ownership	Homogeneous and stable institutional market ownership
Web content and other electronic health care information	Presentation based on professional criteria	Presentation based on institutional standards
Certification	Professional certification of health care workers, services, and institutions	Institutional certification by network affiliation
Market dynamic	External	Internal
Values	Professional (example: American Medical Association)	Citizenship (example: British National Health Service)
Principle	Professional norms, Hippocratic oath	System performance effectiveness: universal service and citizen equality; social contract
	**Model: Free Markets**	**Model: Business Contracts**
Structure:	Open Internet network	Proprietary network (WAN/LAN)
Architecture	Mixed, with autonomous agent systems	Federation
Gateways	Search engines	Corporate gateways
Integration	Dynamic associative	Federative business process (business transactions)
Access control	By individual consumer choices and availability of products and services in the market	By collective choices and network structures
Authority	Decentralized and deprofessionalized, with individual consumer participation	Managerial, with local hierarchies governing institutions and consumer organizations
Ownership	Diverse and dynamic market ownership	Diverse and stable corporate market ownership
Web content and other electronic information	Criteria for individual consumer evaluation of Web content and other electronic information developed with consumer participation	Criteria for collective consumer evaluation of Web content and other electronic information by accreditation agencies
Certification	Of products and services by independent evaluators	Of products and services by institutional evaluators, consumer organizations, and accreditation agencies
Market dynamic	Open consumer	Diverse corporate
Values	Consumer (example: WebMD Health - http://www.webmd.com/)	Managerial (example: Kaiser Permanente)
Principle	Responsible self-regulation; emergent norms	Consumer contract and transaction efficiency (cost/benefit)

The efficiency of rationalization in health care markets depends upon the principles of universal access to information and services and the symmetry of information flows among consumers and health care professionals [30,31]. (Symmetry of information flows refers to a consequence of universal access to information: consumers have access to information as do health care professionals. In traditional health care organizations, consumers do not have such access and so are unable to participate in decision making.) Some research reports, for example, that consumer information with participative decision making and risk sharing may reduce demand for expensive medical procedures such as surgery [32].

The Internet and telecommunications infrastructures contribute to control mechanisms of health care management systems through network structures and transaction services. Technological advances extend the classic concepts of markets or clans and hierarchies in economics and organizational theory [33]. Clan control is expressed through norms and standards emergent in organizational behavior on the Internet as well as publication of health care performance data by professional and regulatory organizations [34]. Examples of clan control include codes of professional conduct and codes of ethics governing cyber behavior as well as norms for presentation of Web content and criteria for consumer evaluation of electronic information. On the other hand, network technologies and the Internet give rise to institutional hierarchies of control embedded within the technologies themselves. For example, the Dynamic Authorization Framework for Multiple Authorization Types (DAFMAT) provides a foundation for user-based, role-based, context-based, and emergency authorizations in health care application systems (vs operating systems) as required by HIPAA (US Health Insurance Portability and Accountability Act of 1996) Security Standards [35]. Such structures may be developed to organize enterprise roles such as those identified in organizational hierarchies. Technological control mechanisms may effectively control access to health care, and ensure social or business contract security, confidentiality, and integrity [29].

While in some cases these infrastructures may replace traditional institutional networks, proprietary networks such as WANs (wide area networks) and LANs (local area networks), intranets, and extranets [36] generally extend or complement such traditional structures and enhance federative system integration. Intranets within such traditional institutions may also serve as vehicles for clan or behavioral control processes. Furthermore, proprietary professional or institutional networks may function in parallel to the Internet to offer hierarchical control while at the same time making the system accessible to an extended community. These tightly-linked electronic hierarchies favor the distribution of high quality products and services as well as the adoption of technological innovations throughout the system, but may impede identification of new trading partners [37].

In the centralized social-medicine model, the role of technology may be fundamentally different from that in the free health care market driven by consumer demand. System performance may be related to the internal coherence of Internet-based, market-oriented governance structures and e-commerce transaction characteristics. Relevant governance structures include electronic hierarchies as well as markets [37]. Electronic hierarchies are firm-specific, generally-bilateral electronic linkages controlled by a centralized managerial system as in the model of social medicine, while electronic markets supported by the Internet and telecommunications networks foster competition among multiple buyers and sellers. In the case of behavioral control, autonomous and distributive multiagent architectures contribute to associative system integration including both human and automated actors, while centralized hierarchical control yields structural or business-process integration through federation architectures [38]. As health care systems extend beyond enterprise, institutional, and national boundaries, associative system integration tends to characterize the evolutionary growth process of functional complementation, while federative architecture emerges in the process of enterprise consolidation [39]. Extensive health care systems may exhibit varying degrees of overlap or redundancy among their associated or federated organizational components. Consistent with open systems theory, such redundancy may contribute to health care system flexibility and adaptability [40].

Table 1 summarizes models describing health care management systems within market dynamics dominated by supply or demand: (1) professional covenants, (2) national constitutions, (3) free markets, and (4) business contracts, governed respectively by professional, citizenship, consumer, and managerial values. These models are not mutually exclusive but reflect ideological diversity in health care [29].

The next sections of the paper will present a case-analysis research methodology used in this study and case analyses of the British National Health Service (NHS) and the California-based Kaiser Permanente (KP) health maintenance organization, selected to validate the national constitution and the business contract models of health care management respectively.

## Comparative Case Analysis

The research methodology used for this study is comparative case analysis. Case analysis is particularly appropriate to health care services research for a number of reasons. The complexity of health care management is increasing, linking diverse subsystems in ever-larger "mega-systems." Such organizational networks, including alliances and partnerships and their underlying ideologies in the health care sector, are extremely difficult to track and measure. System structures and processes are also rapidly changing as a function of technological innovation and economic globalization, rendering model identification more difficult and variable quantification less relevant. These tendencies contribute to the usefulness of case research methods for analysis and assessment of complex health care management systems within their social, economic, and cultural contexts [41-43].

Table 1 presents the health care system models under consideration: the national constitution based on citizenship values, and the institutional business contract model based on managerial values. The organizations to be analyzed are selected according to their classification in these models; the NHS is an example of the national constitution model, while Kaiser Permanente illustrates the business contract model. Each model represents a distinct configuration of characteristics within an institutional and market context. The analytical strategy appropriate to this comparative study is pattern analysis, where patterns of empirical observations are compared to a predicted or theoretical pattern [42]. The model dimensions describing dominant market dynamics (supply and demand) and control processes (clans and hierarchies) are defined as the independent variables, while the characteristics of each model of health care governance specify nonequivalent dependent variables. Multi-trait observations serve to validate the patterns identified in the models. Multiple sources of evidence through a variety of data collection techniques further serve analysis of convergence among different kinds of evidence to validate a single pattern dimension through triangulation [43]. Sources of data used in this study included published research reports from various sources, Internet sites of the health care sector including the organizations under study, and Internet sites of the telecommunications industry. The Web and network structures of NHS and Kaiser Permanente were also analyzed to validate model patterns.

The holistic level of analysis is the health care system and its context [44]. The matrix of health care models in Table 1 (in [44]) guided the identification of evidence within each model to show interrelationships among model dimensions: network architecture and control, information gateways, process and structural integration, access, form of authority, centralization of hierarchy, information quality control, and certification, as well as the values underpinning the institutional health care system.

### National Health Service (NHS)

The British National Health Service (NHS) was created in 1948 to provide health care to all British citizens without regard for their ability to pay for services. The population of the United Kingdom (UK) is now estimated to be nearly 60 million people [45]. The mission of the NHS is to promote the highest level of physical and mental health for all citizens by prevention of ill health, diagnosis and treatment of injury and disease, and long-term care of the chronically ill or disabled. The NHS is publicly funded, accountable to Parliament, and managed by the Department of Health [46] through 8 regional offices. It is responsible for setting, implementing, and evaluating public health policies [47]. The NHS employs nearly 1 million people and requires more than £50 billion for its annual operations. Supported by the Department of Health, local health authorities determine health needs and allocate government funding for services provided by NHS hospital trusts and primary care agencies, while special health authorities, such as the National Blood Authority, provide national services. Hospital treatment is arranged through general practitioner's referrals, except in the case of emergencies. There is also a network of NHS walk-in centers where any citizen can access services and advice. In 1999 a special health authority, the NHS Information Authority [48], was created to develop a national Internet and telecommunications infrastructure for electronic health records, an electronic library of health-related knowledge, and service delivery to all citizens.

National Service Frameworks (NSFs) [49] are being developed by the NHS to improve the quality of services and care offered to patients, to reduce the individual and collective burden of certain conditions, to reduce associated health care inequalities, and to assure uniform standards of quality care throughout the system. An important tenet of these policies for chronic disease management is the empowerment of the patient to participate in health care decision making through information, education, and partnership with health care providers [50,51].

The criteria for definition of National Service Frameworks priorities are relevance to the health care policy agenda; significance in terms of mortality, morbidity, disability, or cost; public concern; care complexity; existence of evidence base for clinical and/or managerial improvements; and need for innovation and service reconfiguration. The aims of this program are to define and implement standards and care models for priority services and care groups, and to elaborate performance measures for systematic reviews conducted by the Commission for Health Improvement [52]. The areas so far identified for framework development include cancer, coronary heart disease, renal failure, diabetes, and mental illnesses. Priority populations include children, the aging, and those suffering from long-term disabilities or conditions threatening their independence and quality of life.

The NHS is currently undergoing reorganization according to The NHS Plan presented to the British Parliament by the Secretary of State for Health by Command of Her Majesty in July, 2000 [53]. This plan is being implemented with a strategy centered on information content, delivery, and technology [54-57]. The major infrastructural axes of the NHS Plan are embodied in:

- NHS Direct [58], which offers services on the Internet that are accessible on a 24-hour basis to all citizens of England. These services provide quality-controlled health information and advice and contribute to more active patient participation in care.
- The National Electronic Library for Health [59] and the Electronic Library for Social Care [60], including NHS-authenticated access to the Cochrane Library [61], which promotes evidence-based medical practice and continuous health care-professional learning as well as patient information. The National Electronic Library for Health also provides links to medical-research review services such as the Data Base of Abstracts of Reviews of Effectiveness (DARE) at the NHS Centre for Reviews and Dissemination, University of York [62].
- Infrastructures which are being developed by the NHS for maintenance of electronic health records in support of patient care. Strategies being considered are NHSnet access through NHS Direct and smart card technologies. The objective is to develop electronic records for every citizen by the year 2005.
- NHSnet [63], which is a secure wide area network for the NHS. It is available from 2 service providers: British Telecom [64] and Cable & Wireless Communications [65]. NHSnet, or the NHS Intranet, is the largest WAN of its kind in Europe. NHSnet provides communication and information services similar to the Internet as well as a dedicated NHS network guaranteeing service and availability, and a secure environment for health care services and information. The services available on NHSnet include: electronic mail service currently provided by Syntegra [66], electronic data interchange (EDI) linking general practice and hospital computer systems, access to NHSweb for affiliated health care professionals, remote support by system suppliers for general practice systems, connectivity to the Internet through secure gateways, authorized Web site hosting (for example, local health authorities), and online patient booking directly into hospitals.

Within the NHSIA (NHS Information Authority), the Healthcare Modelling Programme [67] makes available business models of health care and modelling expertise for use by the NHS, and helps to ensure the internal consistency of central information initiatives. The main product of the Healthcare Modelling Programme is the NHS Healthcare Model (HcM), a business model of NHS activity for use primarily in development of other products including informatics standards, terminology structures, computer applications, and business process designs. Parts of this generic model are available on the Internet and freely licensed to prospective users. Related efforts focus on development and implementation of international communication standards including HL7 (Health Level Seven) [68] and SNOMED (Systematized Nomenclature of Medicine) [69]. The NHS Healthcare Model embodies internal NHS experience in clinical application development and is being used as a guide for implementation of HL7 and as a tool for NHS collaboration in elaboration of future versions of HL7. While HL7 is seen as critical to interoperability of future health care communication standards, a compatible business model needs to be developed to motivate its acceptance throughout the NHS system, particularly in the UK primary-care Clinical Information System (CIS) market. For instance, aspects of HL7 dedicated to representation of insurance and financial transactions are highly specific to US health care systems and inapplicable to the NHS. Current HL7 adoption rates appear to be closely associated with the presence of international companies in domestic British health care markets [70].

An important debate concerns the strategy for NHS networking, particularly the role of the Internet. The Internet would offer ubiquitous access to information necessary to link (through secure, encrypted connections) general practitioners and patients to medical records and clinical information [71,72]. The sustainability of the NHSnet network technology is also an important consideration [73]. A variety of connections link affiliated organizations to NHSnet: public switched telephone networks (PSTN) with modems and ID (identification) security cards, integrated services digital network (ISDN) connections, and fixed-link leased lines and routers. A continuing barrier to NHSnet connection is the diversity of computing systems in general practice organizations and their rate of obsolescence. This problem may be solved by the introduction of national standards for systems suppliers known as Requirements for Accreditation (RFA). However, few systems suppliers are able to conform to the required code of connectivity [74]. The NHS Information Authority offers technical advice and support concerning all aspects of telecommunications infrastructure and connection for NHS organizations including trusts, health authorities, general practices, and third-party affiliates. As the primary link between users and suppliers, NHSnet plays a critical role in maintaining network security standards. Applications for network connections from third-party organizations are processed by NHSnet based on sponsorship by an NHSnet member organization and must be accepted by one of the service providers, British Telecom or Cable & Wireless Communications. Thus linkage to NHSnet is validated for content and the security features of the connection, with final approval granted after technical audit by one of the service providers [75]. The NHS is integrated into national and European programs for development of grid-computing infrastructures under the auspices (among others) of the UK government's Office of the e-Envoy [76], the UK Research Council's e-Science Programme Grid Technical Advisory Group [20] and the British Joint Information Systems Committee (JISC) [77].

Another issue under consideration in the future development of NHSnet and information technology applications in general is the use of proprietary or open-source software. The use of proprietary software demands development of norms and standards for interoperability and the costly maintenance of software licenses, while open-source software can be programmed and adapted according to the needs of the enterprise [78]. The two strategies are very different in the organizational cultures and financial resources necessary to support them [79]. Proprietary software may be supported with outside resources while open-source software requires specialized personnel and programmers within the enterprise to adapt and maintain organization-specific systems. According to some observers, the NHS is unlikely to mobilize the resources necessary to manage open-source software.

Quality control of information and services offered through the telecommunications infrastructure of the NHS is assured by internal processes of standardization, accreditation, and data-driven tracking and assessment. The integrity of the institutional network is protected by technical audit performed by the WAN service providers and NHSnet-member sponsorship of new affiliates. Clinical practice is supported by research organizations and information sources validated through their linkage to NHS Web sites. The challenge is to evaluate care in terms of a community-based assessment rather than the services rendered to an individual patient requesting care [72,80].

The information-led market dynamics of the NHS system promote evidence-based management, governance, and clinical practice for quality monitoring and improvement throughout the system. At the foundation of the model are the integrative core processes of governance, research, and development [81-85]. Institutions created to support clinical governance include the NHS Clinical Governance Support Team (NCGST); the National Institute for Clinical Excellence [86], addressing clinical content; and the Commission for Health Improvement [52], focusing on NHS organization performance. Institutions supporting primary care include the General Practice Research Database [87] and Primary Care Information Services (PRIMIS) [88], engaging about 15% of practices in extraction of data and diffusion of benchmarking information. Although these structures are in place, some research has shown that in the case of the NHS (as in the United States), impact of published clinical outcomes data remains minimal for reasons including credibility, timeliness, awareness, and lack of accountability [89]. These institutions are accessible through the Internet to the public, patients, and health care professionals, thereby achieving broad dissemination and contributing to information symmetry among actors in NHS health care markets. The Centre for Health Information Quality [90], now part of the Help for Health Trust [91], further promotes evidence-based medicine and the production of quality health information for the involvement of consumers as active partners in health care.

Clinical governance has been defined for the NHS as a framework for the development of the organizational capabilities required for sustainable, accountable, patient-oriented, quality-assured health care service delivery integrating health care infrastructures and services delivered within the system through 3 main types of information: (1) health care guidelines, policies, and treatment programs, (2) data descriptive of the care delivered, and (3) data descriptive of the clinical governance system itself [83]. Important issues associated with this definition of clinical governance are the processes of data codification and analysis for translation into useful knowledge as well as the essential contributions of research and development to health care quality [85].

These considerations are particularly important in light of the planned transition from primary care group development in close association with health authorities to independent commissioning agency [92]. This transition will have significant implications for network infrastructures supporting the NHS in terms of information dissemination and outreach to determine population needs. Features of the commissioning process include user involvement, long-term health objectives, conciliation of regulatory requirements with population preferences, and contractor service obligations to the public. These features emphasize relationship management and marketing in contrast to a focus on transactions. Institutional structures will probably lose their boundaries to become synapses linking functions of the health care nervous system in service of individual and community needs. The Internet culture of free and universal access to health care information and services is more consistent with the explicit mission of the NHS than the proprietary, transaction-oriented culture found in many US health care organizations, including Kaiser Permanente, the large California-based health maintenance organization which is the subject of the case analysis presented in the next section of this paper.

### Kaiser Permanente

The organization now known as Kaiser Permanente was founded in 1933 by Dr. Sydney Garfield as a prepaid health plan for workers on a construction project in southern California. In 1938 it was extended to a group practice prepayment plan for Grand Coulee Dam construction workers and their families. By 1945 its programs were available for public enrolment, and Kaiser Permanente is now the largest not-for-profit health maintenance organization in the United States, with more than 8 million members in 9 states and the District of Columbia managed in 7 regional structures. These structures include 3 types of organizations in close cooperation: (1) Kaiser Foundation health plans contracting with individuals and groups to provide medical services through hospitals and medical groups, (2) Kaiser Foundation hospitals owning and operating facilities and services and sponsoring educational and research activities, and (3) Permanente medical groups, partnerships, or physician corporations, fully responsible for providing or arranging regional medical services [9].

Kaiser Permanente requires subscribers to make monthly prepayments depending upon age, sex, and marital status of adult subscribers and number of children to be covered. In addition, subscribers pay fees depending upon the specific services such as office consultations received. Kaiser Permanente also offers programs to groups and businesses. Kaiser Permanente is a competitive group-practice HMO (Health Maintenance Organization) whose performance depends on individual and collective choices of affiliation in US health care markets. Physicians generally receive a salary while some portion of their remuneration may reflect service quality, or financial performance of health plans or medical groups. Plan subscribers are encouraged to choose the personal primary care physician responsible for supervising general care and referrals to special services offered within regional organizations. While the personal physician is characterized as the medical authority to prescribe care, many common services and treatments (including all nonemergency surgical procedures) require pre-certification to determine if the service requested by the patient is medically necessary, conforms to accepted medical practice, and should be covered by Kaiser Permanente. The patient, not the physician, is responsible for informing the health plan management of emergency care, initiating requests for services, and responding to service refusal.

The KP enterprise is an affiliation between two distinct organizations, the Kaiser Permanente Health Plan, an administrative and managerial entity, and the Permanente Medical Group of physicians. Telecommunications network applications and medical information technology innovations have been introduced in HMO operations in telemedicine, for example, in teledermatology, teleradiology, and retinal screening of diabetic patients [93]. Since the mid-1990s, the organization has developed strategies for elaboration of Internet applications on 3 axes: (1) provider knowledge requirements through the Permanente Knowledge Connection (PKC), (2) customer needs through KPOnline, and (3) a shared database. There are 5 major portals associated with the KP health care infrastructure of which the members' portal, KPOnline [94], and the Kaiser Permanente public site [95] are accessible directly through the Internet. Three of the portal sites are accessible only through the KP Intranet: the Permanente Knowledge Connection for clinicians, the KP employee site, and a vendor portal for e-commerce and supply-chain content [96].

The KP Care Management Institute (CMI) [97] created in 1997 is responsible for the Permanente Knowledge Connection and its database. The mission of the Care Management Institute is to create, implement, and evaluate effective and efficient care management programs based on the organization's clinical experience, research, data, and synthesis of information about the best clinical practices. Some research suggests that treatment for acute care in chronic disease populations may account for as much as 80% of hospital stays and 55% of emergency room visits. Care management programs are designed to reduce the costs associated with acute-care incidence and chronic-disease management, through preventive strategies aimed at logical groupings of member populations suffering from priority chronic diseases including diabetes, asthma, congestive heart failure, coronary artery disease, and depression. Characteristics of these diseases are high treatment cost, high incidence in the general population, and existence of a research base for medical practice [98]. Economies of scale and scope continue to motivate the choice of a national framework for care management programs: elimination of program duplication, harmonization of regional efforts, rapid diffusion of innovations across regional organizations, generation of comparable clinical and managerial data throughout the health care system, and improved ability to partner with national health care purchasers, accrediting agencies, and other national stakeholder organizations. Some of the difficulties in implementing such programs on a national scale include variance in resources available in regional organizations and lack of integration of contracted facilities outside Kaiser Permanente [98].

The Permanente Knowledge Connection is a network-based application to support shared access to Care Management Institute content regarding clinical best practices identified throughout the regional organizations. The national intranet connects national and regional databases of best practice information where regional and local offices develop procedures for validation and inclusion of information. This design integrates local authority and national needs for centralized structure. Local market areas are offered assistance to develop Web sites conforming to national KP standards. The goals of the national intranet strategy are to identify procedures for the efficient production and diffusion of information, to facilitate and fund the diffusion of information from best practice sites to other sites throughout the Permanente Knowledge Connection, to develop standards for Web information development, to provide national training for developers, to support research and development on enterprise information and communication, and to develop cost-benefit measurement tools to assess Web technology investments [99]. The information linked in the databases is accessible and searchable through the national intranet Web site thus minimizing redundancy. The Permanente Knowledge Connection offers a system for tracking continuing medical education credits, access to text books and journals, and open and closed discussion and work groups.

The major issues to be addressed in managing the Permanente Knowledge Connection are metadata search capabilities, "push" (that is, supply-driven market dynamics dominated by health care institutions) technologies for delivery of information more specifically useful to care providers, and extending access to affiliated care providers. Related to push information strategies are the goals of computerization of all KP physicians' offices with access to the Permanente Knowledge Connection. In December, 1999, 40% of KP physicians were equipped with desktop computers [93]. An important question to be resolved is the proprietary nature of practice guidelines and the extent to which such information should be freely shared with affiliated providers and the public on consumer-oriented Web sites. One approach is to consider the information nonproprietary while protecting the tools for practice implementation. Ethical issues of privacy also need to be considered, such as performance evaluation of providers' medical-information searches as continuous learning or as evidence of inadequate knowledge and training. Increasing use of the Permanente Knowledge Connection is expected to result in improved knowledge management and health care education, more productive patient-provider interactions, and improved economies of scale with minimized duplication of effort.

KPOnline, the members-only consumer Web site is a 3-tiered system that interacts with legacy systems through an intermediate object layer [93]. The main purpose of the site is to provide a service for interacting with KP members as an alternative to telephone calls and office visits. The service includes health learning materials, communication capabilities, and information about Kaiser Permanente. Members may do research on health concerns through the drug and health encyclopedias, complete a personal health assessment, or browse links to other Internet Web sites. Members may also communicate with KP staff or with other members. For example, advice nurses and pharmacists provide answers to routine questions, and discussion groups with the participation of KP staff members offer opportunities to share experience on a variety of health-related themes, including the Web site itself. Kaiser Permanente's strategy is to offer members attractive services as a foundation for more valuable interactions, contributing to perceptions of self-efficacy and for patients' responsibility in decision making regarding their own health care [100]. Monitoring discussion groups creates an opportunity to collect data concerning member needs and satisfaction. KP peer discussion moderators are trained to create an atmosphere of trust, to inform participants without directly offering medical advice, and to facilitate mutual support among members. The objective of this strategy is to avoid the legal difficulties created in offering online advice to an anonymous audience.

Development of the national intranet has proceeded from the design of local networks in 13 semiautonomous regions with independent data centers and a variety of network configurations, to integration of national operations. Each region of the Kaiser Permanente structure controlled its own information technology resources, resulting in some system duplication [93]. Various networking standards were in use at different locations, making communications between systems and networks difficult. Kaiser Permanente started building the foundation for its national information technology (IT) strategy by selecting standardized technology for all its LANs and consolidating 13 regional data centers into 2 national centers. The national network was designed to support Clinical Information System data, Internet and intranet applications, nationwide e-mail, and telemedicine/teleradiology applications. While the networking foundation of the information technology strategy was put into place, Kaiser Permanente began designing national applications, starting with its National Clinical Information System (NCIS) in the fall of 1998, with deployment to be completed by 2004. Increased network bandwidth will allow KP clinicians to offer customized care to members in person, or via telephone, the Internet, or e-mail.

Adoption of health care standards, in particular HL7 and SNOMED, is of critical importance to the National Clinical Information System strategy. Kaiser Permanente has collaborated with the College of American Pathologists to develop the SNOMED terminology, a foundational component of the evolving National Clinical Information System. Experience in the Southern California region in translating clinical practice guidelines into XML (Extensible Markup Language) has shown that a common set of metadata, for instance based on the Dublin Core [101], needs to be identified before standardization of document structure at the national level [102,103]. Kaiser Permanente has also participated in projects focused on the development of computational grids for high-speed, wide-area, data-intensive computing [104-106]. In the United States, integration of grid infrastructures poses significant challenges to identifying shared tools and resources as well as a business model for management and operation of a service delivery system based on shared responsibility where corporate business models, such as that of Kaiser Permanente, may be considered proprietary.

In the US market, 90% of managed care organizations are evaluated for consumer information by the National Committee for Quality Assurance (NCQA) [107] based on the same performance indicators (the Health Plan Employer Data and Information Set - HEDIS [108]). These measures include effectiveness and accessibility/availability of care; satisfaction with the experience of care, health plan stability, use and cost of services, informed health care choices, and general health plan descriptive information. Quality control in US health care markets has been assured by governmental and independent accreditation agencies, and new agencies are being introduced to address information and telecommunications technologies in health care. The US Health Insurance Portability and Accountability Act of 1996 (HIPAA) [109] has mandated regulations that govern privacy, security, and electronic transactions standards for health care information related to electronic transactions and privacy. These regulations will require major changes in how health care organizations handle all facets of information management, including reimbursement, coding, security, and patient records. In addition, the Electronic Healthcare Network Accreditation Commission (EHNAC) [110], an independent, not-for-profit accrediting body, provides independent peer evaluation of an organization's ability to perform at industry-established levels. EHNAC Security Accreditation will be appropriate for most institutions under the jurisdiction of HIPAA security regulations including clearinghouses, transaction processors, value-added networks (VANs), payers, providers and provider management organizations. However, accreditation for compliance with HIPAA security regulations will also be assured by other accrediting bodies such as the Joint Commission on Accreditation of Healthcare Organizations (JCAHO) [111] and the National Committee for Quality Assurance.

The case of Kaiser Permanente must be considered in the wider context of managed care in the United States and California. Kaiser Permanente is a group health maintenance organization (HMO) working with exclusive, multi-specialty groups of physicians. Other types of HMOs include those that contract with independent practice associations (IPA HMOs), with other medical groups (network HMOs), as well as those that employ staff physicians (staff HMOs). Managed health care has grown rapidly in California where in 1999, 17 million people representing 52% of the state's population were enrolled in 38 HMOs [112]. Some research has shown that HMOs mark up their health care premiums less in competitive markets; however, it is not clear what effect this has on quality of health care [113].

In California, a number of structural problems related to the growth and diversity of managed health care have emerged in the health care industry: inability to select efficient medical groups due to the objective of inclusiveness, resulting lack of competition among medical groups, lack of incentives for physician loyalty or investment in the health care system, and redundant as well as contradictory rules and procedures [112]. In this consumer-led market, there is little consensus regarding the contractual definition of medical necessity, and the decision-making roles of institutional purchasers of health plans are subject to wide variation. Other problems related to market dynamics and the association between medicine and business involve the pharmaceuticals industry. Mass media marketing of prescription drugs and conflicts of interest among pharmaceutical companies, physicians, and medical schools are of particular concern [114-120].

Solutions to these structural problems would likely require more centralized regulation and control of health care organizations inconsistent with the US health care market ideology of free individual or collective consumer choice of physicians and service providers. On the other hand, information and telecommunications infrastructures may contribute to reduce administrative redundancy and increase transaction efficiency in ways already emergent on the Internet [121]. Some examples include data collection and management for research and quality control, standardized auditing and credentialing applications, and general administrative services. See, for example, the California Information Exchange (CALINX) [122] for health care data standardization and exchange, and HealthScope [123] for health care evaluation services focusing on health plans, hospitals, medical groups, and doctors. These services are managed under the auspices of the Pacific Business Group on Health [124], a health care purchaser coalition.

Telecommunications networks may also facilitate direct contracting of services to bypass entirely health care management organizations and to delegate decision making and control to medical groups and physicians, although this tendency will be shaped by regulation governing health care and market responses of traditional health care organizations to their virtual competitors [125,112]. Where health care regulation is determined at the state level, emergence of large buyer and provider organizations will be slowed [126], and the scalability of a large system will be hindered. The fragmented US health care environment further complicates multiple-payer, transaction-oriented systems.

### Case Comparison

The foregoing case analyses of the British National Health Service and the California-based Kaiser Permanente health maintenance organization are summarized in Table 2 showing the comparison of two health care system models, the *National Constitution*(NHS) and *Business Contract*(KP), developed on dimensions guiding this study.

**Table 2:** A comparison of health care system models

	**National Health Service (NHS)**	**Kaiser Permanente (KP)**
System model	National Constitution	Business Contract
Health care market	UK: supply (push)	US: demand (pull)
Market actors	Collective: community	Individual: consumer
Transaction	Relational	Financial
Performance principle	Collective care effectiveness	Individual cost efficiency
Payer	Single-public	Multiple-public and private
Membership	Citizen-birthright Universal access	Consumer-subscription Controlled access
Values	Individual citizenship Collective governance	Consumer financial responsibility Collective management
Integration	Clinical governance	Business process integration
Hierarchy	Centralized institutional authority Decentralized general-practitioner community-practice authority	Centralized managerial authority Decentralized regional authority
Networks	Public proprietary (WAN/LAN) Internet convergence	Private proprietary (WAN/LAN) Internet divergence
	Long term institutional federation (3-year contracts) with associative clinical-process integration	Corporate federation with dynamic associative business-process integration
	Institutional and professional information gateways	Corporate information gateways
Health care provider network	NHSnet	Permanente Knowledge Connection
Consumer-oriented Web site	NHS Direct	KPOnline
Software development and licensing	Proprietary/open-source debate	Proprietary
Standards	Customization/internal adaptation	Standardization/external interoperability
Market ownership	Closed institutional ownership	Open corporate ownership
Quality control	Internal: institutional certification by network affiliation WHO international performance rating Evidence-based practice guidelines Data-based clinical and institutional evaluation	External: accreditation by independent agencies and competitive ratings by consumer organizations Evidence-based practice guidelines Activity-based management Cost-based measures

Both organizations were founded in the 1940s to facilitate access to health care services. These health care systems, founded on very-different ideological premises, share the strategic objective of building telecommunications and Internet infrastructures to reduce health care costs and improve the quality of products and services delivered to patients. Differing market dynamics - "push" (market dynamics dominated by health care institutions) and "pull" (market dynamics dominated by consumer demand) - consistent with the National Constitution (NHS) and Business Contract (KP) models, have significant effects on the configuration of such infrastructures. In the case of the British NHS, the integrative process of governance focuses on collective stakeholders' interests and on flows of information among market actors and institutions. In the case of the California-based health maintenance organization Kaiser Permanente, the integrative process of management focuses rather on financial transactions and evaluation of their cost/benefit efficiency.

The relationship marketing and management model of the NHS places less emphasis on discrete transactions and emphasizes long-term collaboration and contracting. NHS resources are more broadly accessible on the Internet for patient and public information consistent with citizenship values as well as the principles of evidence-based medicine, universal access, and the empowerment of the system user. Such resource availability also facilitates user participation in policy debates and health care decision making as well as related clinical risk sharing. On the other hand, the KP model builds its competitive advantage on the proprietary nature of its products and services, particularly the tools and methods applied to health care delivery. Protection of core KP competencies is consistent with an ideology of managerial values and proprietorship affecting networks, information resources, and software tools. An example of the difference between the two cultures is expressed in the development of clinical and business process decision models. Such models are proprietary tools in the KP culture, while the NHS has a publicly-accessible Internet Web site presenting the flow charts of patient treatment and related business processes, and offering a free license to prospective users of the model [67].

Both health care systems are involved in the development of appropriate SNOMED and HL7 standards. At the NHS, the internal health care model has long served the national infrastructure, and offers a basis for evaluation of SNOMED and HL7 standards adopted primarily where international health care companies are present in internal markets. These national standards also serve to benchmark key areas where international standards need to be adapted to the NHS context. At Kaiser Permanente on the other hand, development of these standards provides a foundation for integrating a diverse national health care organization, making possible a national intranet.

In both cases there is emphasis on data warehousing on the Internet for clinical decision making and evaluation of health care services, while at the NHS, there is further reference to system-wide need for access to tools for data analysis, including algorithms and methods for calculation of national benchmarks to facilitate comparison of local performance on clinical indicators [83]. In the US context, data such as the Health Plan Employer Data and Information Set (HEDIS) collected by the National Committee for Quality Assurance presents standardized indicators for comparative analysis of competitive health plan performance relating health care outcomes to cost. While these tendencies appear to suggest some convergence between the two models and their associated information cultures, it is important to emphasize that such infrastructures serve processes of governance within the NHS, while in the case of Kaiser Permanente they serve competition and the proprietary tools of health care management.

Based on the comparative case analysis, the next section of the paper presents some conclusions, questions and recommendations for researchers and policy-makers in the health care sector.

## Discussion

This comparative case study of two large health care organizations, the British National Health Service and the California-based Kaiser Permanente health maintenance organization has shown how telecommunications and the Internet with other information technologies contribute infrastructures for interactive, integrated, and user-oriented or community-oriented services, as well as governance and resource allocation depending upon organizational forms of control and market ideologies. Information is the foundation of future health care management systems in both cases, including evidence-based medical practice and data-driven health care governance.

The study offers evidence to validate alternative models of health care governance: the national constitution, and the enterprise business contract. This evidence also suggests important questions for health care policy makers as well as for researchers in telecommunications, organizational theory, and health care management. These questions are:

- The public sector NHS and not-for-profit private sector Kaiser Permanente offer services in health care markets dominated respectively by the dynamics of supply (push) and demand (pull) respectively. An important question for future development of information strategies and telecommunications infrastructures in the two organizations is the role of the Internet relative to proprietary network structures (WANs and LANs). Some critics of the NHSnet suggest that secure, encrypted Internet connections would better serve the objectives of the system, particularly to satisfy the need for ubiquity and the integration of patient access. The information culture of NHS also seems more open than does the proprietary culture of Kaiser Permanente. How will such networks and their information cultures coevolve in the future? How will national, community, and ideological boundaries be affected in industry structures and regulation? How will international communication standards, data ownership and intellectual property influence model development? How will open-source and proprietary software serve these models?
- A very important difference between the two systems under study is the mechanism for payment; the NHS is motivated by the principle of universal health care with a single payer and service of collective, community-assessed needs; while Kaiser Permanente is founded on the ideology of individual choice and a diverse multiple-payer system. In the US health care market, some research has shown that the number of financial transactions in the sector is increasing faster than the dollar amount of such transactions, with the consequence that the industry could eventually be "strangled" by the administrative demands of the system [127]. As much as 50% percent of US HMO costs may be associated with administration, defining a priority for development of e-business models applicable to health care and technologies for tracking and automating integrated health care business processes [121,128]. In addition, cost-effectiveness analysis (CEA) related to the transaction-based health care model creates liabilities subject to costly jurisprudence, defining medical necessity and the standard of managed care [129]. The performance ranking published by the World Health Organization [7] suggests that UK health care expenditures are more effective than US expenditures in the sector. How can clinical and business process integration incorporate activity-based management and cost-based measures of performance [130]? Will integrated business processes with network architectures improve the efficiency and effectiveness of the multiple-payer system?
- The processes of diversification and deprofessionalization of health care information, products, and services in markets increasingly driven by consumers raise issues concerning quality control and the ethics of the health care system [82]. The American experience and the case of Kaiser Permanente have demonstrated some of the dysfunctions resulting from the predominance of managerial over professional values. How can managerial and professional values be better reconciled through clinical and business process integration to address efficient system performance and health care rationalization?
- Consumer participation and free health care market dynamics rely on the aggregate of individual choice in governance of health care systems with respect to development of health care models and efficiency of products and services offered by the system. How can individual consumer choices contribute to the ethic of sustainable and equitable health care? What methodologies can be applied to identify and respond to collective community health care needs rather than limiting service to patients who request care individually [131]? How can individual and system requirements be reconciled with the support of telecommunications technologies and the Internet?
- The analyses suggest diverse paths to health care performance, while trends suggest integration among provider, clinician, and patient roles as well as tools for health care communication, clinical decision support, evidence-based medical practice, and performance assessment [132-134]. These changes further contribute to the breakdown of boundaries among medical research, education, and practice as well as between related disciplines such as medical informatics and health care systems management [135-139]. What are the best strategies to ensure integration of differing health care disciplines, cultures, and ideologies? How can international telecommunications and Internet governance through the Internet Corporation for Assigned Names and Numbers (ICANN) [140] contribute to broader system integration?
- The research methodology of the World Health Report [7] has been developed to facilitate comparative analysis of diverse national health care systems, while the Health Plan Employer Data and Information Set (HEDIS) is designed by the US National Committee for Quality Assurance for evaluation of managed health care plan performance. Some researchers suggest that complexity science and system dynamics modelling would be useful to the study of complex health care systems [141]. What theoretical frameworks, as well as qualitative and quantitative methodological approaches, would contribute to definition and study of global health care system value and performance?

This qualitative comparative analysis has shown the significant extent of diversity in health care delivery systems and the critical contributions of the Internet and telecommunications technologies to health care infrastructures. Conclusions and recommendations for future inquiry will contribute to understanding of health care system evolution and formulation of public policy to manage ideologically-diverse markets in the emerging global health care economy.

## Abbreviations

EHNAC: Electronic Healthcare Network AccreditationCommission
HIPAA: US Health Insurance Portability and AccountabilityAct of 1996
HL7: Health Level Seven
HMO: Health Maintenance Organization
KP: Kaiser Permanente
LAN: Local Area Network
NHS: British National Health Service
WAN: Wide Area Network
